# Supplementary material for: Mapping rehabilitation pathways after cardiac surgery: Identifying key points for patient involvement and gaps in care
Source: PLoS One. 2025 Dec 30;20(12):e0324401. doi: 10.1371/journal.pone.0324401 (PMC12752942; doi:10.1371/journal.pone.0324401)
Supplement: S2 File — Guidance for Reporting Involvement of Patients and the Public in Research -Short Form (GRIPP2-SF). (PDF) [file pone.0324401.s002.pdf]

Guidance for Reporting Involvement of Patients and the Public in Research -Short Form (GRIPP2-SF)

Section and topic	Item	Reported on page no/line no(s).
1: Aim	Report the aim of PPI in the study	7/114-115
2: Methods	Provide a clear description of the methods used for PPI in the study	S3 File
3: Study results	Outcomes—Report the results of PPI in the study, including both positive and negative outcomes	S3 File
4: Discussion and conclusions	Outcomes—Comment on the extent to which PPI influenced the study overall. Describe positive and negative effects	S3 File
5: Reflections/critical perspective	Comment critically on the study, reflecting on the things that went well and those that did not, so others can learn from this experience	S3 File
