# Supplementary material for: Mapping rehabilitation pathways after cardiac surgery: Identifying key points for patient involvement and gaps in care
Source: PLoS One. 2025 Dec 30;20(12):e0324401. doi: 10.1371/journal.pone.0324401 (PMC12752942; doi:10.1371/journal.pone.0324401)
Supplement: S3 File — Matrix of involvement of patient partners in the research process. (PDF) [file pone.0324401.s003.pdf]

Matrix of involvement of patient partners in the research process. Figure is adapted from: Rodkjær et al. (2024)

| **Research steps and phases** | | | **Purposes and processes** | **Roles of patient research partners** | | | | |
|---|---|---|---|---|---|---|---|---|
| | | | | **Active listener** | **Informant** | **Advisor** | **Collaborator** | **Decision maker** |
| **Steps** | **Preparation** | Identification of research topic | The overall research project was designed as work packages as part of a larger study aiming to improve cardiac rehabilitation after surgery. Funding was based on this project.<br>The research project and its organisation were presented (Meeting 1) and the patients partners asked clarifying questions. | X | | | | |
| | | Prioritizing of research questions | The research questions were discussed (Meeting 1). Questions were asked and feedback was given based on patient partners experiences of recovery after cardiac surgery and particiaption in cardiac rehabilitation. The research focus was confirmed and refined with minor language revisions. | | X | | | |
| | | Development of study design | The background for the study, the cross-sectional and multidiciplinary setting was presented to provide contextual background for the project. The use of term ”rehabilitation” and the metro map for llustration of different pathways were discussed as well as how professional stakeholder perspectives could be captured. (Meeting 1+2). No replacement for ”rehabilitation” was found. | X | X | | | |
| | **Conduction** | Project administration | The information material for informants, consent forms and interview guide were reviewed and changed based on written feedback (Email+Meeting 2).<br>Expectations and collaboration between researcher and patient partners were agreed upon and one more female partner were recruited (Meeting 1). Content of Minutes were adjusted according to comments (Meeting 2) | | | | X | |
| | | Recruitment of participants | The recruitment plan and identification of key stakeholder were discussed. The inclusion criteria were changed to include another rural municipality at Meeting 2.<br>Representation of participants for individual interview with different illness courses, sociodemgrafic background were emphasized by patient partners. | | | | X | |
| | | Data collection | The data collection process and researcher's experiences were shared and patient partners complemented data by sharing their experiential knowledge of the patient pathway to comfirm and add to findings. An additional focus on children as relatives and the need for psychological support were raised (Meeting 2) | | | X | | |
| | **Finalization** | Analysis and interpretation of data | Continous workshops were held to interpret data and develop themes (Meeting 2-4) .<br>Identification of problem and need for changes, the purpose of intervention and suggestions for relevant components for intervention were applied to a logic model for a prototype of intervention (Meeting 3-4). The guiding principles for the next research phase were discusssed (Meeting 4). | | | | X | |
| | | Dissemination of results | The final metro maps were co-designed with patient partners on content and layout prior to publication (meeting 2-4).<br>A wriiten lay language publication were in collaboration with researchers and patient partners (Meeting 4)<br>Indication of interest in contributing to conference presentation (Meeting 4). | | | | X | |
